# Sprint Mechanical Characteristics of Female Soccer Players: A Retrospective Pilot Study to Examine a Novel Approach for Correction of Timing Gate Starts

**DOI:** 10.3389/fspor.2021.629694

**Published:** 2021-05-28

**Authors:** Jason D. Vescovi, Mladen Jovanović

**Affiliations:** ^1^Faculty of Kinesiology and Physical Education, Graduate School of Exercise Science, University of Toronto, Toronto, ON, Canada; ^2^Faculty of Sport and Physical Education, University of Belgrade, Belgrade, Serbia

**Keywords:** mono-exponential function, maximum acceleration, maximum sprint speed, power, force

## Abstract

The purpose of this study was to compare model estimates of linear sprint mechanical characteristics using timing gates with and without time correction. High-level female soccer players (*n* = 116) were evaluated on a 35-m linear sprint with splits at 5, 10, 20, 30, and 35 m. A mono-exponential function was used to model sprint mechanical metrics in three ways: without a time correction, with a fixed (+0.3 s) time correction, and with an estimated time correction. Separate repeated-measures ANOVAs compared the sprint parameter estimates between models and also the residuals between models. Differences were identified between all modeled sprint mechanical metrics; however, comparable estimates to the literature occurred when either time correction was used. Bias for both time-corrected models was reduced across all sprint distances compared to the uncorrected model. This study confirms that a time correction is warranted when using timing gates at the start line to model sprint mechanical metrics. However, determining whether fixed or estimated time corrections provide greater accuracy requires further investigation.

## Introduction

The assessment of sprint mechanical properties has become popular since a simple method for estimating force, power, and mechanical efficiency was recently published (Samozino et al., 2016; Morin et al., 2019). The outcomes from using this model have potential value for sports scientists by helping identify limitations of short sprint performance as well as to evaluate return to play for injured athletes (Mendiguchia et al., 2014; Morin and Samozino, 2016; Haugen et al., 2019). To date, the literature provides descriptions of sprint mechanical characteristics of male (Buchheit et al., 2014; Samozino et al., 2016; Morin et al., 2019; Edwards et al., 2020) and female (Jiménez-Reyes et al., 2018; Haugen et al., 2019, 2020b; Marcote-Pequeno et al., 2019) athletes for a wide range of sports, but variation in the hardware used to capture sprint performance could influence the modeled kinetic parameters.

The use of force plates is considered the gold standard for assessing mechanical properties of sprinting; however, there are logistical and financial restrictions to capturing the profile of an entire sprint with force plates (Samozino et al., 2016; Morin et al., 2019). Radar and laser technology are more commonly used field-based methods by researchers (Buchheit et al., 2014; Jiménez-Reyes et al., 2018; Marcote-Pequeno et al., 2019; Edwards et al., 2020) but not readily accessible or practical for most practitioners working in sports. To efficiently assess sprint ability within a team setting, the majority of practitioners use timing gates positioned at various distances. Some researchers have incorporated timing gates for sprint testing (Buchheit et al., 2014; Haugen et al., 2019, 2020b) and used the split times to subsequently model force-velocity properties (Samozino et al., 2016; Morin et al., 2019).

The vast majority of practitioners evaluating sprint qualities of athletes use timing gates. There is an inherent limitation when using timing gates to estimate sprint mechanical factors because of the lag time between the first instance of force generation and when the timing gates are initially triggered (start of sprint timing). This lag time results in overestimated parameter estimates for several of the derived metrics (e.g., force, power). In an attempt to resolve this issue, a fixed time correction (+0.5 s) has been recommended (Haugen et al., 2019, 2020b) but not always applied in the literature when using timing gates (Buchheit et al., 2014; Rakovic et al., 2018; Haugen et al., 2020a). Interestingly, the mean difference in duration between timing gates and a block start for 40 m sprint time was +0.27 s (Haugen et al., 2012), but the fixed time correction based on this evidence was nearly two times greater (Haugen et al., 2019, 2020b). Therefore, although a time correction is warranted when using timing gates to avoid errors in estimated kinetic variables, care should be taken when applying one that may be too large that could potentially have the opposite effect (e.g., underestimate power, force). Additionally, implementing a fixed time correction implies that all individuals require an identical correction. Individualizing the time correction is also possible by including it as an estimated parameter within the current model (Samozino et al., 2016; Morin et al., 2019). A recently published study was the first to apply this approach during on-ice sprints with hockey players (Stenroth et al., 2020). However, researchers and practitioners should avoid the assumption that outcomes from male hockey players sprinting on the ice can be directly applied to female athletes sprinting on turf.

Therefore, the purpose of this pilot study was to estimate force-velocity profiles (Samozino et al., 2016; Morin et al., 2019) for female soccer players using timing gates and compare outcomes from three models: without a time correction, with a fixed (+0.3 s) time correction, and with an estimated time correction.

## Materials and Methods

This was a retrospective analysis using a subset of existing data from high-level female soccer players from the United States (*n* = 116, 23.6 ± 2.4 yr, 167.4 ± 6.4 cm, 62.3 ± 7.0 kg) (data from a randomly selected portion of players was used in exploratory analysis and not included in the current study). Ethics approval was provided by an institutional review board, and all athletes signed consent prior to participation. The protocol for assessing linear sprint speed has been described previously (Vescovi, 2012, 2014, 2016). Briefly, all athletes performed a standardized warm-up (~15 min) that included general exercises, such as jogging, shuffling, multidirectional movements, and dynamic stretching exercises. Infrared timing gates (Brower Timing, Utah) were positioned at the start line and at 5, 10, 20, 30, and 35 m at a height of ~1.0 m. The sprint distance and splits were chosen to enable maximal speed to be achieved and assessed. Participants stood with their lead foot positioned ~5 cm behind the initial infrared beam (i.e., start line). Only forward movement was permitted (no leaning or rocking backward), and timing started when the laser of the starting gate was triggered. This start technique eliminates the potential for a “flying” or “rolling” start. The best 35-m time and all associated split times were kept for analysis. The assessment of linear sprints using infrared timing gates does not require familiarization (Moir et al., 2004).

### Sprint Modeling

Short sprints have been modeled using a mono-exponential function (Equation 1.1) (Furusawa et al., 1927), which has become recently popularized (Samozino et al., 2016; Clark et al., 2019). Equation (1.1) represents the function for instantaneous horizontal velocity (*v*) given time (*t*) and two model parameters:

(1) $$\begin{array}{r} v\left(t\right)=MSS\times \left(1-e^{-\;\frac{t}{\;TAU}}\right) \end{array}$$

The parameters of Equation (1.1) are maximum sprinting speed (MSS = m/s) and the time constant (TAU). Mathematically, TAU represents the ratio of MSS to maximum acceleration (MAC = m/s/s) (Equation 1.2):

(2) $$\begin{array}{r} MAC=\frac{MSS}{TAU} \end{array}$$

For split times, distance is the predictor, and time is the outcome variable; thus, Equation (1.1) becomes

(3) $$\begin{array}{r} t\left(d\right)=TAU\times W\left(-e^{\frac{-d}{MSS\times TAU}}-1\right)+\frac{d}{MSS}+TAU, \end{array}$$

where (*W*) in Equation (1.3) represents Lambert's W function (Goerg, 2020).

When using timing gates, a time correction is required because of the lag time between the first instance of force generation and when the timing gates are initially triggered. Without accounting for this lag time the model estimates are inaccurate. Equation (1.3) becomes

(4) $$\begin{array}{r} t\left(d\right)=TAU\times W\left(-e^{\frac{-d}{MSS\times TAU}}-1\right)+\frac{d}{MSS}+TAU\\ \;-time\;correction \end{array}$$

The time correction in Equation (1.4) can be provided as a fixed correction that is selected a priori (Haugen et al., 2012, 2020a), or it can be estimated within the model along with TAU and MSS parameters. The current study implemented a fixed (+0.3 s) time correction as well as an estimated time correction. The fixed correction duration chosen was lower than the previous recommendation based on the following: The average difference for 40-m sprint duration between block starts (capture initial force production) and timing gates is +0.27 s (Haugen et al., 2012). In addition, studies using +0.5 s time correction placed the initial pair of timing gates 60 cm in front of the start line (Haugen et al., 2019, 2020b). Compared with the rolling start, the start procedure in the current study would be expected to result in a shorter duration between initial force production and start time.

Sprint split time data were analyzed separately for each participant with model parameters, force-velocity profiles, and derivative metrics (i.e., force-velocity slope, maximal ratio of force [RFmax], and rate of decrease in RF [DRF]) (Morin and Samozino, 2016) estimated by following previous methods (Samozino et al., 2016; Morin et al., 2019) using the “shorts” package (Jovanović, 2020; Jovanović and Vescovi, 2020) written in R language (R Development Core Team, 2020). The “shorts” package uses non-linear least squares regression implemented in the “nls” function in R (Bates and Watts, 1988; Bates and Chambers, 1992). Both R and the “shorts” package are open-source software.

### Statistical Analysis

Repeated-measures ANOVAs compared each sprint mechanical metric between models. Repeated-measures ANOVAs were also used to compare residual errors between the predicted (modeled) and observed duration for each distance. The average residual error values are reported as the bias. An LSD *post hoc* analysis was used to identify pairwise difference when main effects were observed. Statistical significance was accepted at *p* < 0.05. Cohen's *d* provided the effect size (ES) for pairwise comparisons (Cohen, 1988) and were considered trivial (< 0.2), small (0.2–0.6), moderate (0.61–1.20), large (1.21–2.0), and very large (2.1–4.0) (Hopkins et al., 2009). Pearson product correlations were used to examine the relationship between the estimated time correction value and the associated outcome parameters for maximal acceleration and maximal sprint speed. Data are presented as mean (SD). Statistical procedures were performed using SPSS version 20.0 (SPSS Inc., Chicago, IL, USA).

## Results

Unadjusted sprint durations from the timing gates were 1.20 (0.08) s, 2.00 (0.09) s, 3.39 (0.13) s, 4.71 (0.17), and 5.36 (0.22) s for the 5, 10, 20, 30, and 35 m distances, respectively. The estimated time correction was +0.25 (0.09) s.

Table 1 provides the sprint mechanical parameters and derivative metrics for the three models. All main effects (*p* < 0.001) and pairwise comparisons (*p* < 0.001) revealed differences between the models for each of the variables. Effect sizes between the uncorrected model and both time-corrected models were moderate (*d* = 0.97–1.15 for V0) and very large (*d* = 2.67–4.33 for all other parameters). The effect sizes between the time-corrected models were moderate (*d* = 0.64–0.67 for FV Slope and RFmax) and small (*d* = 0.22–0.59 for all other parameters).

**Table 1:** Sprint mechanical metrics from current study and other studies with female soccer players.

	**TAU**	**F0**	**V0**	**Pmax**	**FV Slope**	**RFmax**	**DRF**
**Study**	**(s)**	**(N/kg)**	**(m/s)**	**(W/kg)**	**(N/s/m/kg)**	**(%)**	**(%)**
**Current**							
No time correction	0.68 (0.12)	11.2 (1.7)	7.61 (0.38)	21.3 (3.3)	−1.48 (0.25)	59 (3)	−13.2 (2.1)
Fixed time correction	1.20 (0.18)	6.6 (0.8)	8.14 (0.53)	13.4 (1.5)	−0.81 (0.12)	46 (3)	−7.6 (1.1)
Estimated time correction	1.10 (0.16)	7.1 (0.9)	8.03 (0.48)	14.2 (1.8)	−0.89 (0.13)	48 (3)	−8.2 (1.1)
**Statistics (*****p*****-value, d)**							
No correction vs. fixed	< 0.001, 3.40	< 0.001, 3.46	< 0.001, 1.15	< 0.001, 3.08	< 0.001, 3.42	< 0.001, 4.33	< 0.001, 3.34
No correction vs. estimated	< 0.001, 2.97	< 0.001, 3.01	< 0.001, 0.97	< 0.001, 2.67	< 0.001, 2.96	< 0.001, 3.67	< 0.001, 2.98
Fixed vs. estimated	< 0.001, 0.59	< 0.001, 0.59	< 0.001, 0.22	< 0.001, 0.48	< 0.001, 0.64	< 0.001, 0.67	< 0.001, 0.55
Marcote-Pequeno et al. (2019) (Radar)		6.3 (0.4)	8.12 (0.44)	12.7 (1.2)		46 (4)	−7.2 (0.5)
Jiménez-Reyes et al. (2018) (Radar)							
Elite/internat		6.5 (0.3)	8.18 (0.47)	13.2 (1.0)			
Semi-prof		6.5 (0.6)	7.60 (0.38)	12.2 (1.3)			
Haugen et al. (2019) (Gates-0.5 s fixed)		~7.6	~8.1	~15.5	~-0.94	~43	~-9.2
Haugen et al. (2020b) (Gates-0.5 s fixed)							
National		7.6 (0.5)	8.1 (0.4)	15.5 (1.3)	−0.99 (0.07)	43 (2)	−8.9 (0.7)
Top		7.5 (0.4)	7.8 (0.4)	14.7 (1.3)	−0.97 (0.06)	42 (2)	−9.2 (0.6)
Junior		7.6 (0.7)	7.8 (0.4)	14.8 (1.3)	−0.97 (0.08)	42 (2)	−9.2 (0.8)

*TAU-time constant; F0-theoretical maximal horizonal force production; V0-theoretical maximal running velocity; Pmax-maximal horizontal mechanical power output; FV slope-force velocity slope; RFmax-maximal value for ratio of force; DRF-rate of decrease in ratio of force with increasing speed during sprint acceleration. There were main effects (p < 0.001) and pairwise differences between all three models for each sprint mechanical variable*.

**Figure 1:**
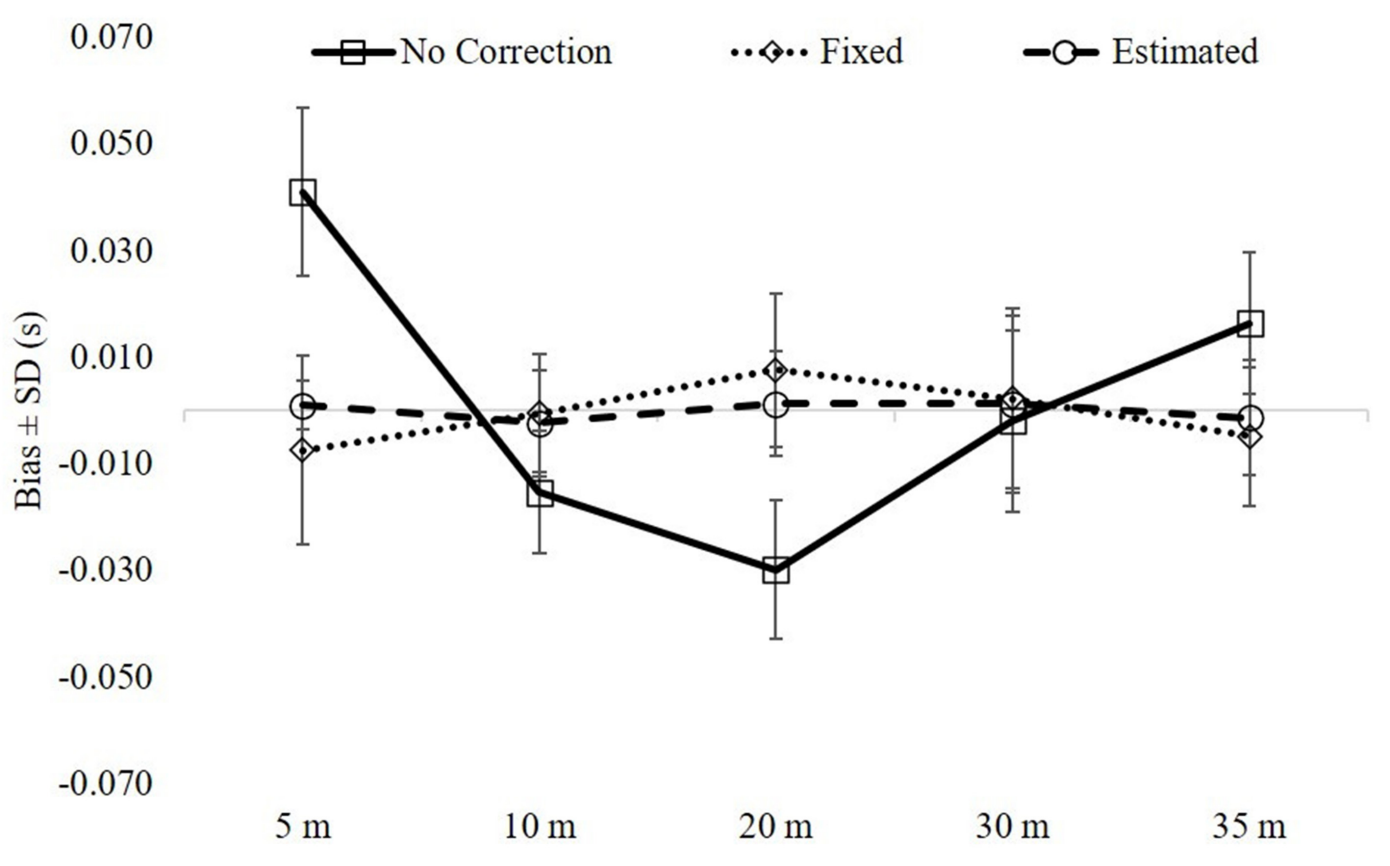
The overall model fit metrics (bias ± SD) grouped by distance. There were main effects (*p* < 0.001) for each distance with differences for all pairwise comparisons between the three models (*p* < 0.001). See text for effect sizes.

Figure 1 includes the bias (SD) for each model grouped by distance. There were main effects (*p* < 0.001) for each distance with differences for all pairwise comparisons (*p* < 0.001). Effect sizes were large to very large for 5–20 m (*d* = 1.2–3.4), small for 30 m (*d* = 0.20–0.24), and large for 35 m (*d* = 1.47–1.62) when comparing the uncorrected model against both models with time correction. Effect sizes between the two models with time correction were trivial to moderate (*d* = 0.06–0.65).

Table 2 shows the maximum acceleration and maximum sprint speed values for the uncorrected, fixed time correction, and estimated time correction models. There was a main effect for acceleration (*p* < 0.001) with differences found for all pairwise comparisons (*p* < 0.001). The effect sizes for the model with no time correction and the other maximal acceleration values were very large and between the time-corrected models was moderate. There was also a main effect for maximum sprint speed (*p* < 0.001) with differences found for all pairwise comparisons (*p* < 0.001). The effect sizes between the model with no time correction and both time-corrected models were moderate, whereas it was trivial between the two time-corrected models.

**Table 2:** Sprint timing metrics.

	**MACC**	**MSS**
	**(m/s/s)**	**(m/s)**
**Models**
No correction	11.3 (1.7)	7.46 (0.36)
Fixed	6.6 (0.8)	7.85 (0.46)
Estimated	7.2 (0.9)	7.77 (0.43)
**Statistics (*****p*****-value, d)**
No correction vs. fixed	< 0.001, 3.54	< 0.001, 0.94
No correction vs. estimated	< 0.001, 3.01	< 0.001, 0.78
Fixed vs. estimated	< 0.001, 0.70	< 0.001, 0.18

*MACC, maximal sprint acceleration; MSS, maximal sprint speed. There were main effects (p < 0.001) and pairwise differences between all three models*.

**Figure 2:**
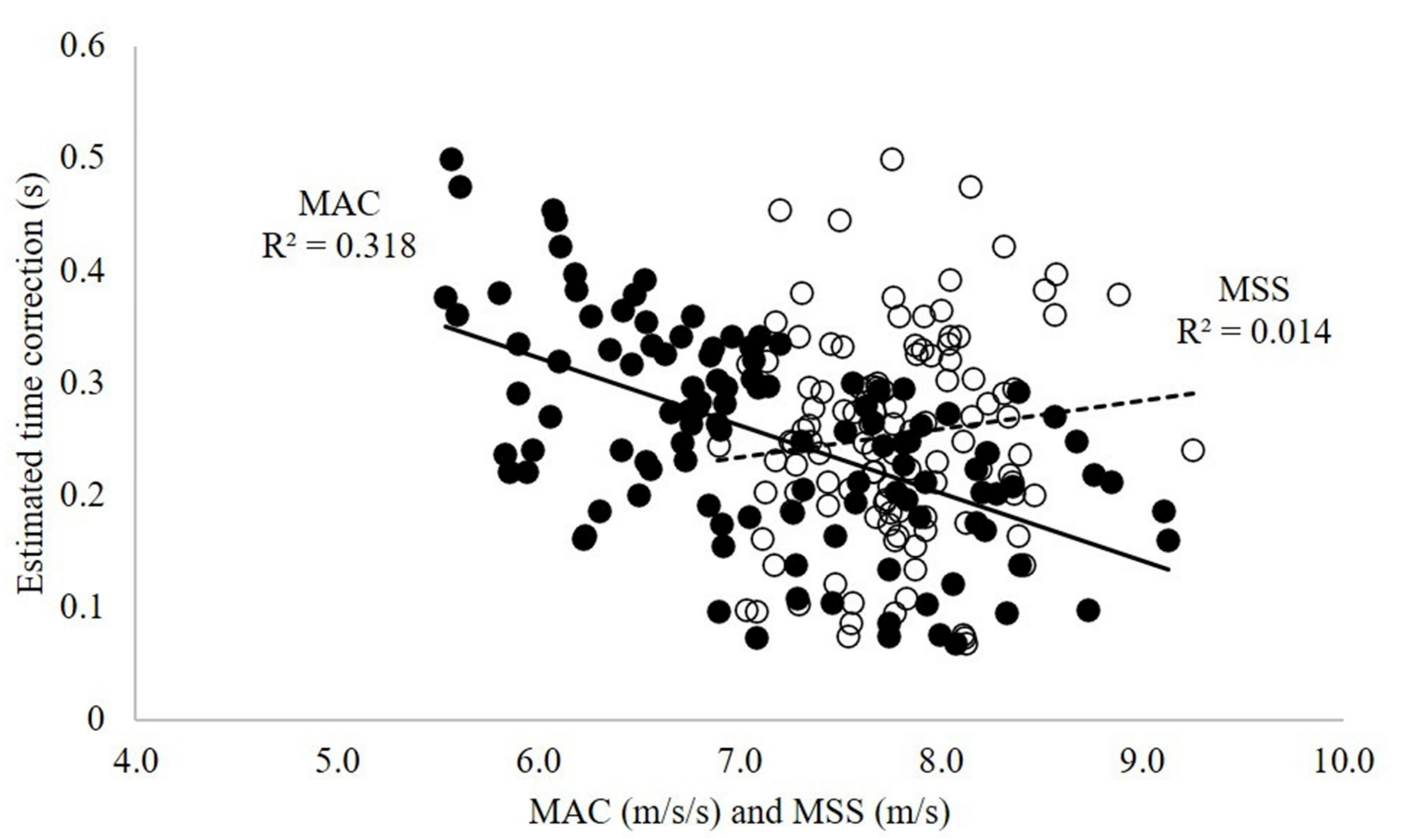
Scatterplot for the estimated time correction value with maximal acceleration (MAC-solid circles, solid line) and maximal sprint speed (MSS-open circles, dashed line).

Figure 2 shows the scatterplots between the estimated time correction value and the corresponding maximal acceleration and maximal sprint speed parameter outcomes. There was a strong linear relationship between the time correction value and maximal acceleration (*r* = −0.564, *p* < 0.001) but no correlation with maximal sprint speed (*r* = 0.119, *p* = 0.21).

## Discussion

The current study extends the findings of other researchers and demonstrates the model with no time correction produced substantially different estimates of sprint mechanical parameters in female soccer players when using timing gates. The outcomes uniquely highlight that fixed (+0.3 s) and estimated time corrections (0.25 ± 0.09 s) improved these estimates, which were better aligned with values previously reported in the literature for similar cohorts. Furthermore, bias was substantially reduced for both time-corrected models.

To obtain accurate estimates from timing gate input with this method (Samozino et al., 2016), the start time needs to be very closely associated to initial force production into the ground. As expected, the uncorrected model outcomes displayed substantially different values compared with the outcomes from both time-corrected models (Table 1). The time corrected models provided estimates that were closely aligned with previous studies reporting on female soccer players (Jiménez-Reyes et al., 2018; Haugen et al., 2019, 2020b; Marcote-Pequeno et al., 2019). The observed differences with studies that also used timing gates is likely a result of the start procedure and time correction that was used. One group of researchers has implemented two types of timing systems and two types of starts (both approaches were simultaneously assessed and demonstrated no differences in 40 m sprint time) (Haugen et al., 2019, 2020b). The first method had a touch pad under the athlete's front foot that would trigger the timing start when released. The second method positioned a single-beam timing gate and the athlete's center of mass 60 and 50 cm in front of the start line, respectively (Haugen et al., 2019, 2020b). A +0.5 s fixed correction was applied in these studies to adjust the triggering of the timing system to “first movement.” Another study with elite female handball players also used a touch pad under the foot to trigger the timing gates but applied no time correction, yet still reported similar sprint mechanical characteristics to other studies that used time correction (F0 = 7.3 ± 0.3 N/kg, V0 = 8.0 ± 0.3 m/s, Pmax = 14.6 W/kg, FV slope −0.91 ± 0.04 N/s/m/kg) (Rakovic et al., 2018). In contrast, players in the current study placed the toes of their front foot 5 cm behind the start and were only allowed to move forward to begin the sprint, thereby reducing the gap between initial force production and the start time. The difference in start technique is the reason for using a smaller fixed time correction (+0.3 vs. +0.5 s). Surprisingly, the mean estimated time correction (0.25 ± 0.09 s) was very similar to the fixed correction as well as the difference reported between block and standing sprint starts (0.27 ± 0.12 s). Taken together, these outcomes highlight that the type of start technique could influence the time correction required and may lend support for using an estimated time correction.

To the authors' knowledge, only one recently published study has included an estimated time correction into this model (termed time shift optimization) (Stenroth et al., 2020). Hockey players performed a 30 m sprint on the ice, and outcomes demonstrated improved intra- and inter-rater reliability for the model using a time shift when evaluating force-velocity profiles. The average time shift reported was +0.14 s, which is smaller than the fixed and estimated time correction in the current study. This could possibly reflect improved sensitivity for capturing the first instance of force production when using video compared with the timing gates in our study. Alternatively, it might represent performing sprints on two different surfaces (ice vs. turf). Also worth noting is that the range of estimated time correction values in the current cohort were all positive (+0.07 to +0.50 s) (Figure 2) and reached as high as the fixed values previously reported (Haugen et al., 2019, 2020b). The negative linear relationship between the estimated time correction values and maximal acceleration highlights that individuals with faster acceleration had smaller corrections. Taken together, these outcomes seem to support the use of methodology-specific time shifts (corrections) estimated on an individual level instead of fixed shifts.

The current study used a method for capturing sprint time that is unique in the literature (i.e., position of the athlete relative to the start line, plus the two time corrections). Therefore, direct comparisons of model outcomes to other studies poses a challenge. Nonetheless, there are some interesting illustrations. A study with U18 boys (radar) demonstrated slower mean 5 m (1.33 vs. 1.20 s), similar 10 m (2.07 vs. 2.00 s) and 20 m (3.35 vs. 3.39 s), and faster 30 m sprint times (4.57 vs. 4.71 s) than the current group of players (Edwards et al., 2020). The resulting modeled outcomes provided greater F0 (8.0 N/kg), V0 (8.85 m/s), and Pmax (17.7 W/kg) values but comparable FV slope (−0.91 N/s/m/kg), RFmax (45%) and DRF (−8.2%) (Edwards et al., 2020) with the current athletes. This was also reflected with a mixed group (male and female) of team handball players using a similar testing approach with timing gates (uncorrected) and showed greater F0 (7.8 N/kg), V0 (8.65 m/s) and Pmax (17.1 W/kg) values, but comparable FV slope (-0.92 N/s/m/kg), RFmax (45%), and DRF (−8.5%) outcomes (Haugen et al., 2020a). Despite recording faster sprint times in the current group of athletes than previously published for high-level female soccer players (timing gates: 10 m = 2.17 ± 0.06 s; 20 m = 3.55 ± 0.11 s; 30 m = 4.84 ± 0.16 s), there were larger values for force, power, FV slope, and DRF reported in the literature (Table 1) (Haugen et al., 2019). It is unclear if this demonstrates that primary sprint mechanical metrics (i.e., force, velocity and power) may be more sensitive to data inputs than other derived metrics (i.e., FV slope, RFmax, DRF) or if the various approaches used (e.g., touch pad start, timing gate start with and without correction, etc.) have a greater influence on the models. Regardless, there were differences between the two time corrected models for all of the variables in the current study, and even though small-to-moderate effect sizes were observed, the differences were greater than previously reported CV% (Morin et al., 2019; Haugen et al., 2020a). Therefore, additional research is warranted to determine which method of time correction (fixed vs. estimated) provides more accurate parameter estimates when using timing gates.

The time constant (TAU) represents the duration it takes a system (in this case an athlete that is sprinting) to achieve 63.2% of maximum (speed). TAU has been thought of as a useful indicator of acceleration with smaller values representing the achievement of maximum sprint speed more quickly and vice versa (Healy et al., 2019). Previous studies with NFL players (Clark et al., 2019) and elite female sprinters (Greene, 1986) reported TAU values between 0.77 and 0.91 s, whereas elite male sprinters have consistently shown values between 1.0 and 1.2 s (Greene, 1986; Healy et al., 2019; Morin et al., 2019). Upon first inspection, this might be perceived as female soccer players having similar acceleration qualities as 100 m Olympic/World Championship male sprinters. However, when taken in context of the duration taken to cover equivalent distances, then the differences in performance over a short sprint becomes evident between the current cohort (20 m = 3.39 s) and male sprinters (20 m = 2.82 s) (Healy et al., 2019). Indeed, it has been shown that smaller TAU values only occurred for faster sprinters after controlling for maximum velocity and is the reason these parameters need to be considered together (Healy et al., 2019). Therefore, maximal acceleration (Equation 1.2) is a better estimate for this sprint quality and should be used instead of TAU.

## Conclusion

The primary outcomes from the current study confirm that a time correction is warranted when using timing gates to estimate sprint mechanical parameters. A limitation of the current retrospective pilot study is that a reference method (i.e., laser, radar) was not used; therefore, additional investigation is warranted to determine whether a fixed time correction or estimated time correction provides greater accuracy when assessing force-velocity profiles from sprints performed on turf. It is likely that the previously suggested fixed time correction (+0.5 s) is too large (Haugen et al., 2019, 2020b) because the mean estimated time correction (+0.25 ± 0.09 s) was closer to the fixed time correction as well as the previously reported difference between block and standing starts (+0.20 to +0.33 s) (Haugen et al., 2012). It may also be possible that methodology-specific time corrections are needed (Stenroth et al., 2020).

Until it can be determined which method offers better estimates for sprint mechanical metrics when using timing gates, it might be prudent (and simpler) for practitioners to utilize a fixed time correction. Keep in mind, the sprint start procedure will influence the value chosen. For example, +0.30 s was used in the current study because of the specific position of the athlete relative to the start line. If the athlete is positioned further behind the start line, then it would be appropriate to use a larger time adjustment value. Practitioners interested in applying the individual estimated time correction can use the “shorts” package specifically designed for this purpose (Jovanović, 2020; Jovanović and Vescovi, 2020).

## Data Availability Statement

The datasets presented in this article are not readily available because of pre-existing legal agreements. Requests to access the datasets should be directed to Dr. Jason Vescovi.

## Ethics Statement

The studies involving human participants were reviewed and approved by York University, Office of Research Ethics. Written informed consent to participate in this study was provided by the participants.

## Author Contributions

JDV was responsible for study design, data collection, data interpretation, writing, and revision of the paper. MJ was responsible for data analysis and modeling, data interpretation, writing, and revision of the paper. Both authors contributed to the article and approved the submitted version.

## Conflict of Interest

The authors declare that the research was conducted in the absence of any commercial or financial relationships that could be construed as a potential conflict of interest.
